# Construction of novel pJRD215-derived plasmids using chloramphenicol acetyltransferase (*cat*) gene as a selection marker for *Acidithiobacillus caldus*

**DOI:** 10.1371/journal.pone.0183307

**Published:** 2017-08-16

**Authors:** Rui Wang, Chunmao Lin, Jianqiang Lin, Xin Pang, Xiangmei Liu, Chengjia Zhang, Jianqun Lin, Linxu Chen

**Affiliations:** State Key Laboratory of Microbial Technology, Shandong University, Jinan, China; TU Bergakademie Freiberg, GERMANY

## Abstract

**Background:**

*Acidithiobacillus caldus*, a Gram-negative, chemolithotrophic sulfur-oxidizing bacterium, is widely applied in bioleaching. The absence of an ideal selection marker has become a major obstacle to achieve high efficiency of the gene transfer system for *A*. *caldus*. Plasmid pJRD215, widely used in *Acidithiobacillus* spp., has severe drawbacks in molecular manipulations and potential biosafety issues due to its mobility. Therefore, finding a new selection marker and constructing new plasmids have become an urgent and fundamental work for *A*. *caldus*.

**Results:**

Effective inhibitory effect of chloramphenicol on the growth of *A*. *caldus* was elucidated for the first time. The P2-*cat* gene cassette, including a chloramphenicol acetyltransferase gene (*cat*) from plasmid pACBSR and a promoter (P2) upstream of the tetracycline resistance gene on pBR322, was designed, chloramphenicol acetyltransferase was expressed in *A*. *caldus*, and the enzyme activity was assessed. A new vector pSDU1 carrying the replication and mobilization regions derived from pJRD215, the P2-*cat* gene cassette and a multiple cloning site from pUC19 was successfully constructed. Compared with pJRD215, pSDU1 had a 27-fold increase in electrotransformation efficiency (30.43±0.88×10^4^ CFU/μg DNA for pSDU1 and 1.09±0.11×10^4^ CFU/μg DNA for pJRD215), better carrying capacity and could offer more convenience for the restriction enzyme digestion. In addition, the generated plasmid pSDU1Δmob, a novel non-mobilizable derivative of pSDU1 lacking some DNA sequences involved in the mobilization process, had increased copy number in *A*. *caldus* and lost its mobility for biosafety considerations. Both pSDU1 and pSDU1Δmob exhibited stable maintenance in *A*. *caldus* within 50 passages. However, further deletion of *orfEF* region involved in regulating *repAC* operon resulted in a negative effect on transformation efficiency, copy number and stability of plasmid pSDU1ΔmobΔorfEF in *A*. *caldus*.

**Conclusion:**

Chloramphenicol was proved to be an ideal selection marker for *A*. *caldus*. Novel plasmids carrying *cat* gene were constructed. The utilization of these vectors will undoubtedly facilitate efficient genetic manipulations and accelerate the research progress in *A*. *caldus*.

## Introduction

*Acidithiobacillus caldus* is a Gram-negative, acidophilic, obligately chemolithotrophic, moderately thermophilic bacterium [1]. It generates energy by oxidizing reduced inorganic sulfur compounds (elemental sulfur, sulfide, sulfite, thiosulfate and tetrathionate) and fixing carbon dioxide from the air [2,3]. As one of the most abundant bacteria in bioleaching tanks of mineral ores, *A*. *caldus* can work cooperatively with other iron-oxidizing bacteria to facilitate bioleaching. In recent years, *A*. *caldus* has attracted researchers’ extensive attention because of its unique physiological characteristics and important applications in the bioleaching industry. Multiple *A*. *caldus* strains were isolated from the environment and the genome sequences were released [4,5], which provided a wealth of information on the genetic composition of this bacterium. However, the cellular functions of most of the putative proteins and the unique sulfur oxidation system of *A*. *caldus* remain unknown.

The gene transfer systems, more specifically, the conjugation system and electroporation system, are powerful techniques in genetic engineering for gene functional studies *in vivo*. Applications of conjugation techniques were reported in the representative species in *Acidithiobacillus* genus, including *A*. *thiooxidans*, *A*. *ferrooxidans*, and *A*. *caldus* [6–9]. In contrast, gene transfer by electrotransformation has been successful in *A*. *ferrooxidans*, and *A*. *caldus* only [10,11]. However, relatively low transfer efficiency has been observed in either conjugation or electrotransformation, which is an obstacle for functional studies in *Acidithiobacillus* genus.

The plasmid pJRD215 derived from RSF1010 has been widely employed in *Acidithiobacillus* genus as the shuttle vector for gene transfer because of its broad host range, the autonomous-replication capability in the host and the antibiotic resistance genes (*kan* and *str*) [12]. However, pJRD215 has several drawbacks when it is used in molecular biology research and genetic engineering in *Acidithiobacillus spp*.: (i) the instability of streptomycin and kanamycin in acidic environment leads to the low transformation efficiency and high false positive transformant yield; (ii) the multiple cloning site (MCS) on pJRD215 contains few recognition sites for restriction enzymes, most of which are not commonly used, resulting in troubles during molecular manipulations; (iii) the large size of pJRD215 (10.3 kbp) limits its carrying capacity and causes severe pressure on the growth of *A*. *caldus* cells; (ⅳ) the mobility of pJRD215 brings biological safety issues in industrial applications. However, the still acceptable transfer frequency and good maintenance of pJRD215 make it the best shuttle plasmid in *Acidithiobacillus* genus so far [13].

The essential replication (*repA-repB-repC*) and mobilization (*mobA*-*mobB*-*mobC*) regions of pJRD215 are derived from IncQ group plasmid RSF1010 [14,15]. RSF1010 is a broad host range vector that can be transferred by conjugation in many bacterial species [16,17]. The genes and loci involved in plasmid vegetative replication and mobilization have been identified in RSF1010. Among the four promoters in RSF1010, P1 and P3 initiate the transcription of *mobA*/*repB*, *mobB*, and, probably, *orfE*-*orfF*-*repA*-*repC* operon. The resulting MobA, together with MobC, auto-regulates the promoters P1, P2 and P3. Promoter P2 is in charge of the transcription of *mobC* in the opposite direction. P4 promoter controlled by OrfF repressor regulates the transcription of the *orfE*-*orfF*-*repA*-*repC* operon [18]. The essential replication proteins RepA, RepB and RepC and replication origin *ori*V control the initiation of DNA replication in RSF1010 [19], and this self-replication property extends the host range of RSF1010 [15]. Meanwhile, plasmid RSF1010 also carries the origin of conjugative DNA transfer (*ori*T) and the three genes (*mobA*, *mobB* and *mobC*) for mobilization purpose [20].

Electrotransformation, as an advanced gene transfer method, provides a convenient and efficient way for introducing exogenous DNA into mammalian cells, plants cells, yeasts, and bacteria [21–24]. Plasmid pJRD215 is the prior choice of shuttle vector for introducing foreign DNA sequences into *A*. *caldus* cells. However, for electroporation, the genes involved in mobilization on pJRD215 become unnecessary. Researchers have shown that the deletion of these mobilization genes in RSF1010 not only increases the copy number of the plasmid, but also ensures the antibiotic safety in applied biotechnology by decreasing the mobility rate of the plasmid [25,26]. Therefore, it is necessary to modify pJRD215 for applications. As *A*. *caldus* is acidophilic, an optimal antibiotic selection marker effective at low pH (pH<2.5) for *A*. *caldus* is required in order to improve the transfer efficiency. For years, kanamycin and streptomycin have been the only two antibiotics used for screening *A*. *caldus* recombinants. However, neither of them is quite effective in liquid Starkey-S^0^ medium (pH 2.0–2.5). Though kanamycin and streptomycin are used in solid Starkey-Na_2_S_2_O_3_ medium (pH 4.8) for selection purposes, the screening efficiency is too low to eliminate the false-positive cells. So, an acid stable selection marker other than kanamycin and streptomycin is needed to improve the gene transformation efficiency in *A*. *caldus*.

The aims of this study are to find the effective antibiotics marker to improve the conjugation and electroporation efficiencies and construct new plasmids for *A*. *caldus*, which will provide useful tools for molecular operations of *A*. *caldus*.

## Results and discussion

### Inhibitory effect of chloramphenicol on the growth of *A*. *caldus* cells

**Fig 1:**
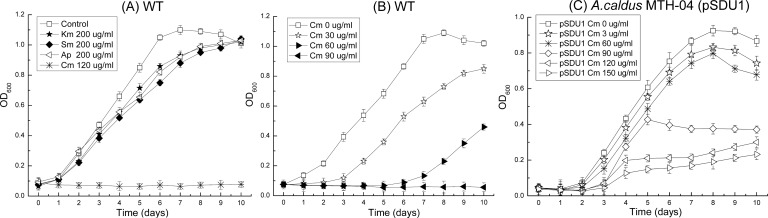
Growth curves of *A*. *caldus* strains grown in liquid Starkey-S^0^ media with different antibiotics. (A) *A*. *caldus* MTH-04 wild type in media with kanamycin, streptomycin, ampicillin and chloramphenicol, respectively. (B) *A*. *caldus* MTH-04 wild type in media with different concentrations of chloramphenicol. (C) *A*. *caldus* MTH-04 carrying pSDU1 in various concentrations of chloramphenicol.

The inhibitory effect of antibiotics on the growth of *A*. *caldus* cells in Starkey-S^0^ liquid media was measured as shown in Fig 1A. Chloramphenicol at a concentration of 120 μg/ml in liquid Starkey-S^0^ media could inhibit the growth of *A*. *caldus* MTH-04 cells completely during the whole test period (data collected every 24 hours for 10 days), while the other antibiotics (ampicillin, kanamycin and streptomycin) had minor inhibitory effects on the growth of *A*. *caldus* MTH-04 cells after 1 day. Furthermore, the growth of *A*. *caldus* MTH-04 cells in liquid Starkey-S^0^ media containing different concentrations of chloramphenicol was tested. As shown in Fig 1B, chloramphenicol at a concentration of 30 μg/mL had a significant inhibitory effect on the growth of cells in the first 3 days; 60 μg/ml chloramphenicol could completely inhibit bacterial growth for 6 days and the inhibitory effect became weaker afterwards; 90 μg/ml chloramphenicol inhibited bacterial growth in the whole testing period (10 days). In addition, no colony was observed on the Starkey-Na_2_S_2_O_3_ solid agar plate containing chloramphenicol (60 μg/mL) within 15 days (data not shown). Taken together, these results suggested that chloramphenicol is suitable for antibiotic selection of *A*. *caldus* cells in both liquid and solid media.

The relatively low pH level suitable for the growth of *A*. *caldus* is an adverse factor for the effectiveness of most of the antibiotics. The optimal pH for the growth of *A*. *caldus* is 2.0–2.5, and the initial pH of liquid Starkey-S^0^ medium is 2.5. As the cells proliferate, elemental sulfur is oxidized to sulphuric acid, and the pH decreases to 0.8 in the stationary phase. Most antibiotics lose their functions in extremely low pH. Therefore, neither ampicillin, kanamycin nor streptomycin exhibits inhibitory effects on the growth of the cells (Fig 1A). Chloramphenicol is one of the most stable antibiotics and is effective in the pH range 0.4–10 [27]. In this study, we found that chloramphenicol at 90 μg/ml can successfully inhibit the growth of *A*. *caldus* in liquid Starkey-S^0^ medium (Fig 1B). Together with the fact that relatively low concentration of chloramphenicol (60 μg/mL) is needed to inhibit the growth of the cells on the solid agar plates, our results suggested that chloramphenicol is a suitable selection marker for molecular researches on *A*. *caldus* cells.

### Construction of the plasmids carrying chloramphenicol acetyltransferase (*cat*) gene

**Fig 2:**
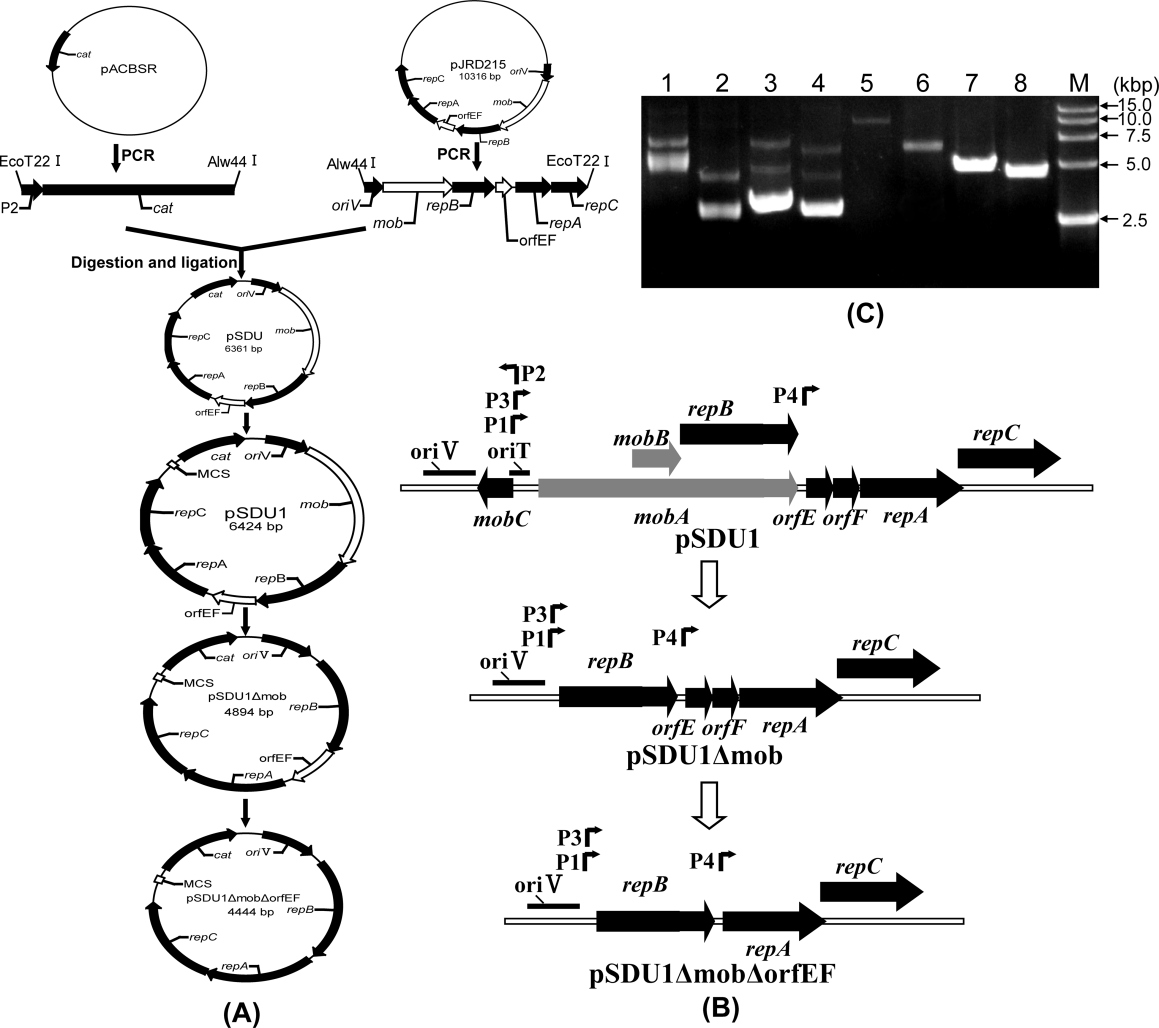
Construction of pJRD215-derived plasmids. (A) Construction processes of pSDU1, pSDU1Δmob, and pSDU1ΔmobΔorfEF. A P2-*cat* cassette was constructed by the fusion of the promoter P2 from the plasmid pBR322 and the *cat* gene from plasmid pACBSR. The generated cassette and the backbone amplified from plasmid pJRD215 were ligated to generate plasmid pSDU. After that, the multiple cloning sites originated from pUC19 were introduced into pSDU to construct the plasmid pSDU1. To construct pSDU1Δmob, the *mobB* gene and a part of *mobA* gene on pSDU1 was removed. Finally, the *orfEF* region was further deleted from pSDU1Δmob, resulting in plasmid pSDU1ΔmobΔorfEF. (B) The detail diagram of the new pJRD215-derived plasmids. (C) Electrophoretic analysis of plasmids. Lane 1, 5: pJRD215; lane 2, 6: pSDU1; lane 3, 7: pSDU1Δmob; lane 4, 8: pSDU1ΔmobΔorfEF; lane 5–8, plasmids digested with *Hind* Ⅲ.

The completely inhibitory effect of chloramphenicol on the growth of *A*. *caldus* made the *cat* gene a suitable antibiotic selection marker for constructing novel plasmids. The detailed constructing processes of the vectors are described in the method section (Fig 2). To ensure the expression of the *cat* gene in *A*. *caldus* MTH-04, the promoter (P2) located upstream of the tetracycline resistance gene on the plasmid pBR322 was fused to the translational start site of the *cat* gene to generate P2-*cat* cassette [28]. The replication and mobilization regions of pJRD215 were used as the backbone of the generated plasmid pSDU1. In addition, pSDU1 also carried the MCS originated from pUC19. Based on the knowledge of the genetic loci responsible for the vegetative plasmid replication and mobilization of plasmid RSF1010 [18, 19], the loci including the *mobB* gene and a part of *mobA* gene on pSDU1 were removed to create a non-mobilizable plasmid pSDU1Δmob and the *orfEF* region was further eliminated from pSDU1Δmob to construct pSDU1ΔmobΔorfEF (Fig 2).

The key characteristics of the constructed plasmids (pSDU1, pSDU1Δmob and pSDU1ΔmobΔorfEF) are as follows. (1) The chloramphenicol acetyltransferase gene was used as the selection marker in these plasmids, which improved the screening efficiency. (2) New multiple cloning sites were introduced into these plasmids, which facilitated efficient genetic manipulations. (3) The backbone of these plasmids was originated from the broad host range plasmid RSF1010, which introduced broad host range and autonomous replication capacity into the newly designed plasmids. (4) The sizes of pSDU1, pSDU1Δmob and pSDU1ΔmobΔorfEF are only 6.4, 4.9 and 4.4 kbp, respectively. Compared with pJRD215 (10.3 kbp), the much smaller sizes of the three new plasmids allowed them to carry larger genetic inserts. (5) The non-mobilizable character of pSDU1Δmob and pSDU1ΔmobΔorfEF, caused by the deletion of *mob* region, enhances the biological safety in real applications.

### Determination of *cat* gene function in *A*. *caldus*

To test the expression of the constructed P2-*cat* cassette, plasmid pSDU1 was electroporated into *A*. *caldus* MTH-04 [11]. Chloramphenicol was used for the screening of electrotransformants. Colonies were picked from the solid plates containing 68 μg/ml chloramphenicol and transferred into liquid Starkey-S^0^ medium containing different concentrations of chloramphenicol. In the end, the plasmid pSDU1 was recovered from the electrotransformants (data not shown). To investigate the inhibitory effect of chloramphenicol on the growth rate of *A*. *caldus* cells harboring pSDU1, the cells were cultured in liquid Starkey-S^0^ medium added by different concentrations of chloramphenicol and the growth was determined by measurement of the optical density (OD_600_). As shown in Fig 1C, with the final concentration of 0, 30, 60, 90, 120 and 150 μg/ml chloramphenicol in the culture broths, the maximum value of OD_600_ of *A*. *caldus* MTH-04 carrying pSDU1 could reach approximately 0.9, 0.8, 0.8, 0.4, 0.3, 0.2, respectively. Cells grew well when the concentration of chloramphenicol in the culture broth was less than 60 μg/ml; 90 μg/ml chloramphenicol obviously inhibited cell growth; and 120 and 150 μg/ml chloramphenicol had severe inhibition effects on the cell growth.

Since the autotrophic *A*. *caldus* is phylogenetically distant from other well-studied heterotrophic bacteria, e.g. *E*. *coli*, many known promoters that work in other bacteria can not initiate the transcription of genes in *A*. *caldus*. To ensure the transcription of chloramphenicol resistant gene in *A*. *caldus*, promoter P2 was used, which was from pBR322 and proved to have transcriptional capacity in *A*. *caldus*. The success of transformant selection on agar plates containing chloramphenicol indicated that the antibiotic resistant gene was expressed and worked well in *A*. *caldus*. Chloramphenicol functions as a bacteriostatic, by inhibiting protein chain elongation during protein synthesis. To protect the cells from being killed by chloramphenicol, the *cat* gene encoding a chloramphenicol acetyltransferase, was introduced into the cells to acetylate chloramphenicol and prevent it from binding to the ribosome. So, the transformants can grow well at 90 μg/ml chloramphenicol while the wildtype cells that do not carry any *cat* gene in their genome will be eliminated. With considerations of the inhibitory effect of chloramphenicol on the growth of both transformants and the wildtype cells, the suitable final concentration of chloramphenicol, 60–90 μg/ml, in broth culture was determined for antibiotic selection purpose.

### Determination of electrotransformation efficiency and frequency

A significant improvement on electrotransformation efficiency and frequency was obtained when chloramphenicol was used as the selective marker. As shown in Table 1, the transformation efficiencies of plasmids pSDU1 and pSDU1Δmob were 30.43±0.88 ×10^4^ and 38.67±0.79 ×10^4^ CFU/μg DNA, respectively, while it was 1.09±0.11×10^4^ CFU/μg DNA for plasmid pJRD215 using kanamycin as the selective marker. Compared with pJRD215, the transformation efficiencies of pSDU1 and pSDU1Δmob increased 28 and 34 times, respectively. Meanwhile, the transformation frequencies of pSDU1 and pSDU1Δmob increased approximately 27 and 34 times, respectively. The results indicated that addition of *cat* gene in the two new plasmids enhanced their transformation efficiencies and frequencies significantly (See Table 1 for detailed information on transformation efficiencies and frequencies). In contrast, plasmid pSDU1ΔmobΔorfEF had relatively lower transformation efficiency and frequency compared with those of pSDU1 and pSDU1Δmob (Table 1).

**Table 1:** Transformation efficiency and frequency of plasmids in electroporation of *A*. *caldus* MTH-04.

	Transformation efficiency^a^ (×10^4^ CFU/μg DNA)	Transformation frequency^b^ (×10^−6^ CFU/cells added)
pJRD215	1.09±0.11	2.55±0.25
pSDU1	30.43±0.88	71.00±2.05
pSDU1Δmob	38.67±0.79	90.22±1.85
pSDU1ΔmobΔorfEF	0.12±0.01	0.28±0.03

^a^ Mean±Standard deviation.

^b^ Mean±Standard deviation.

The increased transformation efficiencies and frequencies of pSDU1 and pSDU1Δmob likely result from the significant inhibitory effect of chloramphenicol on the growth of the wildtype *A*. *caldus* cells. After electroporation, the cells need some recovery time and antibiotics should be added after that time to inhibit the growth of negative transformants, which can increase the ratio of positive transformants to wildtype on selective agar plates. The protein encoded by *orfF* can autoregulate the *repAC* operon by inhibiting transcription from promoter P4 [18]. Mutagenesis of *orfEF* could weaken the feedback from *orfF* to *repAC* operon and in turn, affected the expression levels of *repA* and *repC* in pSDU1ΔmobΔorfEF, so this mutation made the plasmid unstable in *A*. *caldus*. This could possibly explain the significantly reduced transformation efficiency and frequency of pSDU1ΔmobΔorfEF.

### Determination of conjugative transfer frequencies

The mobility of the series of plasmids in *A*. *caldus* was tested by the conjugation and reverse-conjugation as previously described [9]. As shown in Table 2, the transfer frequency of pSDU1 from *E*. *coli* SM10 to *A*. *caldus* MTH-04 had a 3-fold increase compared to that of pJRD215, while no transconjugant carrying pSDU1Δmob or pSDU1ΔmobΔorfEF was obtained. Utilization of chloramphenicol as the selection marker and the smaller size of pSDU1 (6.4 kbp) could contribute to the higher transfer frequency of pSDU1. In the reverse-conjugation, *A*. *caldus* MTH-04 transformants carrying plasmids pJRD215, pSDU1, pSDU1Δmob or pSDU1ΔmobΔorfEF, were used as the donor strains to conjugate with *E*. *coli* C600 recipient strain with the help *E*. *coli* C600 harboring plasmid RP4. The results are shown in Table 2. The transfer frequency of pSDU1 increased slightly compared with that of pJRD215 while the transfer frequencies of pSDU1Δmob and pSDU1ΔmobΔorfEF (less than 10^−7^) were undetectable. The results indicated that the deletion of a 1,636 bp fragment at the initial part of *mobA* gene (Fig 2B) made the plasmids lose their mobility, which improved the biological safety. These results were same with the previous report that RSF1010-derived plasmids with partially deleted *mob*-genes possessed undetectable mobilization frequencies under laboratory conditions [25,26].

**Table 2:** Transfer frequencies of plasmids between *A*. *caldus* MTH-04 and *E*. *coli* C600.

Donor	Recipient	Selection marker	Transfer frequency ^a^^,^^b^
*E*. *coli*	*A*. *caldus*		
SM10 (pJRD215)	MTH-04	Sm^r^	(3.21±1.11) ×10^−5^
SM10 (pSDU1)	MTH-04	Cm^r^	(9.54±1.41) ×10^−5^
SM10 (pSDU1Δmob)	MTH-04	Cm^r^	—
SM10 (pSDU1ΔmobΔorfEF)	MTH-04	Cm^r^	—
*A*. *caldus*	*E*.*coli*		
MTH-04 (pJRD215) and *E*. *coli* C600 (RP4)^c^	C600	Sm^r^	(2.53±1.31) ×10^−6^
MTH-04 (pSDU1) and *E*. *coli* C600 (RP4)^c^	C600	Cm^r^	(3.62±1.28) ×10^−6^
MTH-04 (pSDU1Δmob) and *E*. *coli* C600 (RP4)^c^	C600	Cm^r^	—
MTH-04 (pSDU1ΔmobΔorfEF) and *E*. *coli* C600 (RP4)^c^	C600	Cm^r^	—

^a^ Data are averages of at least three independent experiments.

^b^ Mean±Standard deviation.

^c^ *E*. *coli* C600 (RP4) was used as a helper strain.

—undetected.

### Plasmid copy number of pSDU1, pSDU1Δmob and pSDU1ΔmobΔorfEF in *A*. *caldus*

Two calculation methods (absolute quantification and relative quantification) based on real-time qPCR results were used to estimate the copy numbers of the pJRD215-derived plasmids in *A*. *caldus*.

**Fig 3:**
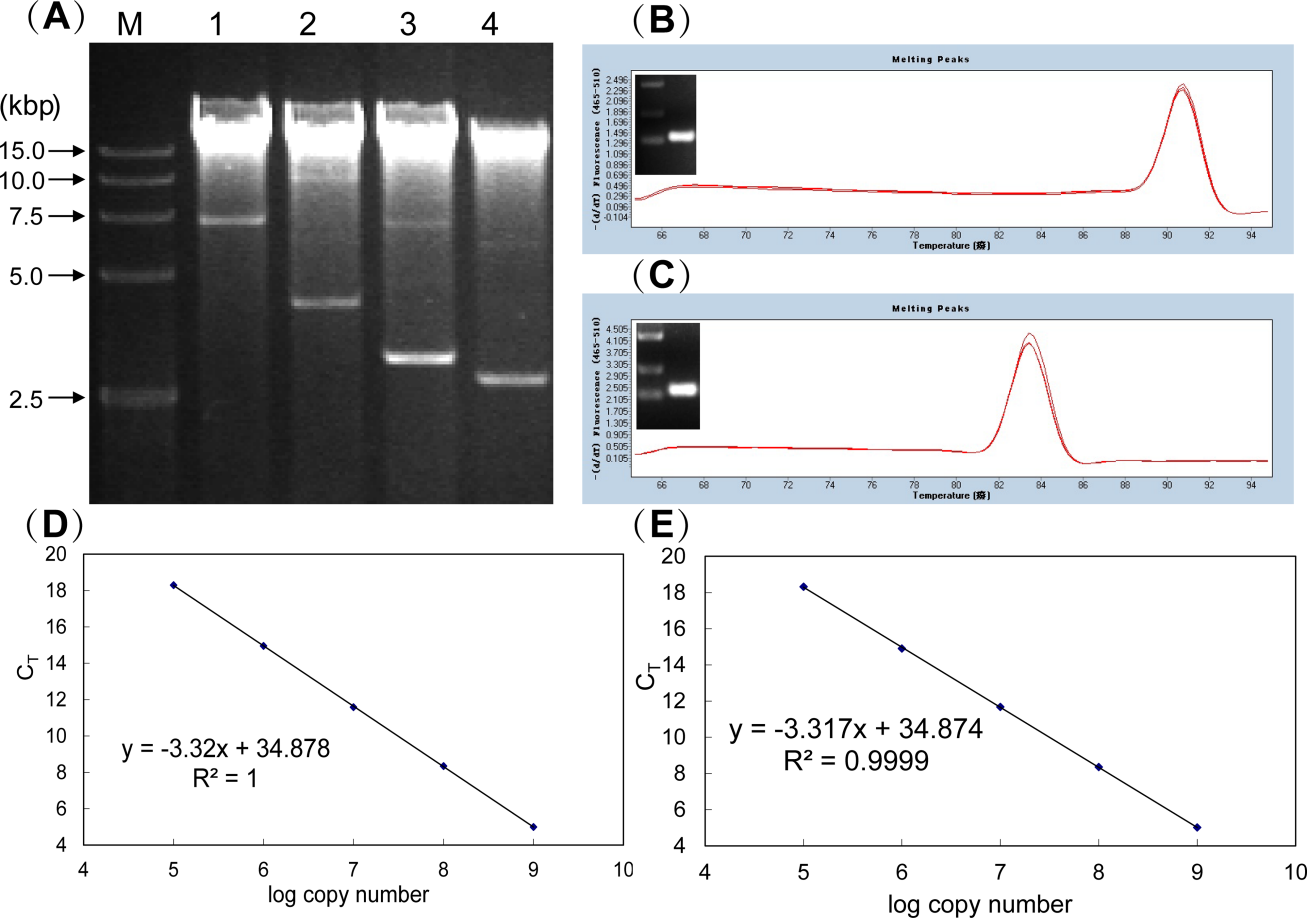
Real-time qPCR experiments to determine the copy numbers of plasmids in *A*. *caldus* MTH-04. (A) Electrophoretic analysis of the total DNA from *A*. *caldus* MTH-04 carrying pJRD215 (lane 1), *A*. *caldus* MTH-04 carrying pSDU1 (lane 2), *A*. *caldus* MTH-04 carrying pSDU1Δmob (lane 3), *A*. *caldus* MTH-04 carrying pSDU1ΔmobΔorfEF (lane 4), respectively. Confirmation of PCR amplification specificities of *tetH*-set (B) and *repA*-set (C) primers. Standard curves and amplification efficiencies of *tetH*-set (D) and the *repA*-set (E) primers.

First, total DNA was purified from four different recombinants of *A*. *caldus* each carrying one of the plasmids (pJRD215, pSDU1, pSDU1Δmob and pSDU1ΔmobΔorfEF) (Fig 3A). Then, two sets of primers were designed specific to the single-copy genes *repA* (from pJRD215) and *tetH* (from *A*. *caldus* chromosomal DNA). The amplification specificities of *repA*-set and the *tetH*-set primers were analysed by both melting curve analysis and gel electrophoresis. Sharp peaks were observed in the melting peak analysis on both sets of primers (Fig 3B), and prominent bands with expected sizes were found in the gel electrophoresis analysis for the real-time qPCR products (Fig 3C). The identities of amplified products were confirmed by sequencing analysis afterwards. These results indicated that non-specific PCR products amplified using these primer sets were undetectable in the analysed temperature ranges. The standard curves for *repA* and *tetH* genes, ranging from 0.5×10^5^−0.5×10^9^ copies/μL, were drawn in Fig 3D and 3E, respectively. Both curves were linear in the tested range (R^2^>0.999) based on the triplicate reactions. The slopes of the standard curves for *repA* and *tetH* were -3.32 and -3.317, respectively. From the slopes, the amplification efficiencies (E) of 1.00 for both *repA* and *tetH* primer sets were calculated in the investigated range and were used for relative quantification. Finally, these two sets of primers were used in real-time qPCR to obtain the C_T_ (threshold cycle) values of *repA* and *tetH* genes in these total DNA samples, and the these C_T_ values were used for further calculations.

Absolute quantification was carried out according to the method described earlier [29]. The absolute copy numbers of *repA* and *tetH* in *A*. *caldus* were determined based on the corresponding standard curves (Fig 3D & 3E), using the C_T_ values. Because *repA* and *tetH* are single-copy genes on pJRD215 and *A*. *caldus* chromosomal DNA, respectively, the copy ratio of *repA* to *tetH* is equal to the plasmid copy number. Thus, the plasmid copy number was then calculated by dividing the copy number of *repA* by the copy number of *tetH*. As shown in Table 3, the copy numbers of pJRD215, pSDU1, pSDU1Δmob, pSDU1ΔmobΔorfEF in *A*. *caldus* were ~10, 11, 28 and 19, respectively (derived from absolute quantification).

**Table 3:** Estimated plasmid copy number (PCN) by absolute quantification.

Plasmid	C_T_ ^a^		Copies^b^ (copies/μl)		PCN^c^
	*repA*	*tetH*	*repA*	*tetH*	
pJRD215	12.47±0.03	15.87±0.02	5.62×10^6^ (1.8%)	5.37×10^5^ (1.4%)	10.45 (3.3%)
pSDU1	12.04±0.03	15.48±0.02	7.55×10^6^ (2.1%)	7.04×10^5^ (1.1%)	10.72 (3.1%)
pSDU1Δmob	9.48±0.03	14.32±0.02	4.46×10^7^ (2.1%)	1.57×10^6^ (1.1%)	28.42 (3.0%)
pSDU1ΔmobΔorfEF	12.37±0.03	16.61±0.03	6.03×10^6^ (1.7%)	3.21×10^5^ (1.8%)	18.82 (3.5%)

^a^ Average±S.D. (n = 3).

^b^ Average (coefficient of variation) (n = 3).

^c^ Average (coefficient of variation) (n = 3).

For relative quantification, *repA* and *tetH* were used as the target and the reference genes, respectively. Because the standard plasmid pJRD215-tetH (i.e, calibrator) had one copy of each *repA* and *tetH* specific sequence, the copy ratio of *repA* to *tetH* in the calibrator should be 1 and the ΔC_T_ of *repA* to *tetH* should be 0. In the experiment, ΔC_T_ of the calibrator was -0.02 with acceptable experimental error compared to the theoretical value (Table 4). The ΔC_T_ of the samples was obtained from the C_T_ of *repA* and *tetH* shown in Table 4. Therefore, the ΔΔC_T_ calculated from ΔC_T_ of the sample and calibrator is equal to the copy ratio of *repA* to *tetH* in the sample. Then the copy numbers of the plasmids were obtained by the formula (1+E)^-ΔΔCT^ [30]. Using the experimentally calculated amplification efficiency (E = 1.00), the plasmid copy numbers were determined using the 2^−ΔΔCT^ calculation. The copy numbers of pJRD215, pSDU1, pSDU1Δmob, pSDU1ΔmobΔorfEF in *A*. *caldus*, calculated from relative quantification, were ~10, 11, 28 and 19, respectively (Table 4). The results of both absolute and relative quantification were identical and reproducible.

**Table 4:** Estimated plasmid copy number (PCN) by relative quantification method.

Plasmid	ΔC_T_ sample^a^	ΔC_T_ calibrator^b^	ΔΔC_T_^c^	PCN^d^
				2^-ΔΔCT^
pJRD215	-3.40±0.05	-0.02±0.05	-3.38±0.05	10.39 (3.3%)
pSDU1	-3.43±0.05	-0.02±0.05	-3.41±0.05	10.66 (3.1%)
pSDU1Δmob	-4.84±0.04	-0.02±0.05	-4.82±0.04	28.26 (3.0%)
pSDU1ΔmobΔorfEF	-4.24±0.05	-0.02±0.05	-4.22±0.05	18.69 (3.5%)

^a^ Average±S.D. (n = 3).

^b^ Calculated from the serial dilutions of the quantitative standard sample used for standard curve construction. Average±S.D. (n = 10).

^c^ Average±S.D. (n = 3).

^d^ Average (coefficient of variation) (n = 3).

The copy numbers of pJRD215 and pSDU1 in *A*. *caldus* (10 per cell) were slightly lower than the reported value of RSF1010 in *E*. *coli* (12 per cell) [31]. The differences in the copy number in the two host bacteria is probably caused by their differences in growth characteristics: the chemolithotrophic growth of *A*. *caldus* and the heterotrophic growth of *E*. *coli*. The main structure of pSDU1 is similar to pJRD215, both carrying the replication and conjugation regions, thus the copy number of the two plasmids in *A*. *caldus* was basically the same.

The deletion of certain functional genes (*mobB* and part of *mobA*) on pSDU1 resulted in the obvious increase in the copy number of pSDU1Δmob in *A*. *caldus*. In pSDU1Δmob, the distance from promoters P1 and P2 to the replication gene *repB* is reduced compared with that of pSDU1. Meanwhile, the inhibitory effect of MobA on promoter P3 is eliminated in pSDU1Δmob. All of these possibly led to the increase in transcription rates and expression levels of *repB* and its following genes *repA* and *repC*, which would in turn increase the copy number of plasmid pSDU1Δmob. Our results are in accordance with the recent findings that the copy number of RSF1010-based plasmids increased significantly when the *mob*-genes are partially removed [25,26].

The copy number of pSDU1ΔmobΔorfEF decreased significantly in comparison to that of pSDU1Δmob in *A*. *caldus*. Further deletion of *orfE* and *orfF* genes from pSDU1Δmob probably got rid of the inhibitory effect of *orfF* gene product to P4 promoter or affected the transcription of *repA*, *repC* and *repB* so as to influence the replication and stability of the plasmid. This possibly explained the reduced copy number of pSDU1ΔmobΔorfEF in *A*. *caldus*.

### Maintenance of the plasmids in *A*. *caldus*

**Fig 4:**
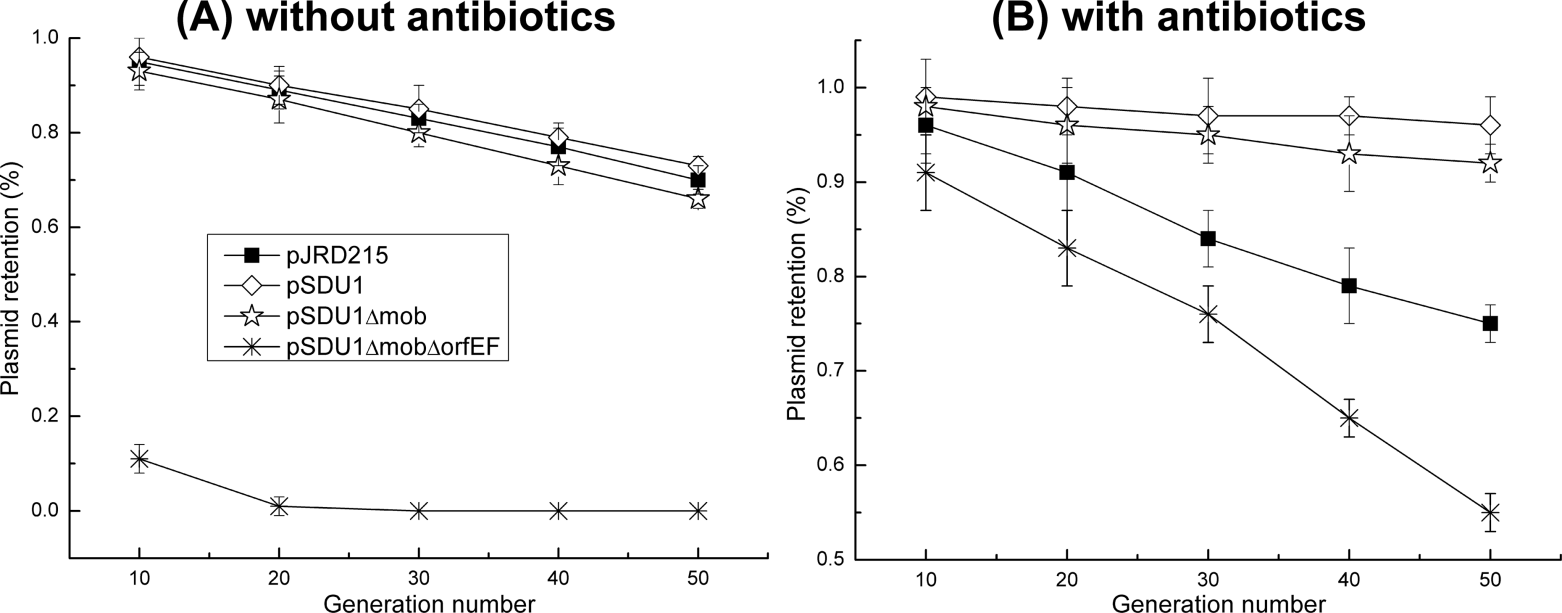
Maintenance of the plasmids in *A*. *caldus* MTH-04 cultivated in liquid Starkey-S^0^ media during serial passages. (A) without antibiotics; (B) with antibiotics, streptomycin 200 μg/ml, chloramphenicol 60μg/ml.

Plasmid stability analysis in *A*. *caldus* was carried out as described in the method section. After serial passages for approximately 50 times, the plasmid retention ratios were calculated and shown in Fig 4. When *A*. *caldus* cells passed 50 passages in culture broth without any antibiotics, the retention ratios of pJRD215, pSDU1, pSDU1Δmob were 70%, 73% and 66%, respectively. The plasmid retention rate of pSDU1ΔmobΔorfEF after 10-time-passage was approximately 11% and the plasmid was lost after 20 passages. As shown in Fig 4B, since chloramphenicol is stable in Starkey-S^0^ medium, the addition of chloramphenicol into the culture broth increased significantly the retention rates of pSDU1 and pSDU1Δmob to 90% after 50 passages, and the retention rate of pSDU1ΔmobΔorfEF to 50% after 50 passages. In contrast, kanamycin and streptomycin could not increase the retention rate of the plasmid pJRD215. Since these antibiotics are not stable at pH 2.5, they have no inhibitory effects on the growth of the cells.

The deletion of replication and mobilization regions of pSDU1 had effects on the stabilities of the generated plasmids at various degrees. Without the antibiotic selection pressure, there were no significant differences in the retention rates among pJRD215, pSDU1 and pSDU1Δmob (Fig 4B), which indicated that the removed mobilization region was not essential for the plasmid stability in *A*. *caldus*. The significant decline in the retention rate of pSDU1ΔmobΔorfEF compared with that of pJRD215, pSDU1 and pSDU1Δmob in the culture broth with and without antibiotics, suggested the importance of *orfEF* region on the plasmid stability in *A*. *caldus*. Due to the regulatory role of OrfF on the expression of the *repAC* operon, the deletion of *orfEF* may have serious negative effects on the replication of the plasmid resulting in the instability of pSDU1ΔmobΔorfEF in *A*. *caldus*. Even with the addition of chloramphenicol to the culture, pSDU1ΔmobΔorfEF was still lost in most the cells.

## Conclusions

The lack of a suitable selection marker for the gene transfer systems has been a major obstacle for the genetic manipulations in *Acidithiobacillus* genus. In this study, we discovered the significant inhibitory effect of chloramphenicol on the growth of *A*. *caldus*, and constructed a family of pJRD215-derived plasmids harboring the chloramphenicol acetyltransferase gene. Compared with pJRD215, these newly constructed plasmids, pSDU1, pSDU1Δmob and pSDU1ΔmobΔorfEF, are smaller in sizes (≤ 6.4kbp) and carry the *cat* gene and the different multiple cloning sites. Due to the incorporation of the *cat* gene, the electroporation efficiency of pSDU1 had a progressive increase. Further deletion of certain sequences related to mobilization from pSDU1 resulted in the two new derivatives (pSDU1Δmob and pSDU1ΔmobΔorfEF) which were shown to be no longer mobilizable. Thus, this two plasmids could be considered as non-mobilizable vectors, which improved the biosafety in industrial applications. Moreover, there was an approximately 3-fold increase in the copy number of pSDU1Δmob in *A*. *caldus* compared with that of pJRD215. However, the removal of *orfEF* region had negative effects on the transformation efficiency, plasmid copy number and stability, suggesting that the absence of repressor OrfF would impair the plasmid replication.

To sum up, the miniaturized plasmid pSDU1, the higher copy number (in comparison to pSDU1) and non-mobilizable plasmid pSDU1Δmob, both carrying chloramphenicol selection marker, were considered as suitable vectors for *A*. *caldus* studies. In addition, the construction of these new plasmids and the development of electroporation and conjugation techniques together constitute a complete upgraded gene operating system for *A*. *cladus*, which will be useful tools for future functional studies and genetic engineering in *A*. *caldus*.

## Methods

### Bacterial strains and cultivation conditions

The bacterial strains and plasmids used in this study are listed in Table 5. *E*. *coli* was grown in Luria-Bertani broth or Luria-Bertani agar at 37°C [32], with the addition of ampicillin (100 μg/ml), kanamycin (100 μg/ml), streptomycin (100 μg/ml) and chloramphenicol (30 μg/ml) when needed. *A*. *caldus* MTH-04 was grown at 40°C in a liquid Starkey-S^0^ inorganic medium containing ampicillin (200 μg/ml), kanamycin (200 μg/ml), streptomycin (200 μg/ml) and chloramphenicol (60 μg/ml) or on the solid Starkey-Na_2_S_2_O_3_ medium containing kanamycin (100 μg/ml), streptomycin (100 μg/ml) and chloramphenicol (60 μg/ml) when needed [6].

**Table 5:** The bacterial strains and plasmids used in this work.

Strain or plasmid	Phenotype or genotype	Source or reference
Strain		
*E*. *coli* SM10	Km^r^ *thi-1 thr leu tonA lacY supE recA*:: RP4-2-Tc::Mu	[34]
*E*. *coli* C600	*integrated* *thr*, *leu*, *hsd*	Shandong University, China
*E*. *coli* DH5α	F^-^φ80d *lacZ*ΔM15Δ(*lacZYA-argF*) U169 *end A1 recA1 hsdR17*(r_k_^-^,m_k_^+^) *supE44λ-thi-1 gyr96 relA1 phoA gyr96 relA1 phoA gyr96 relA1 phoA*	TransGen Biotech Corp. China
*A*. *caldus* MTH-04	Wild-type	[35]
Plasmid		
RP4	*Ap*^*r*^, *Tc*^*r*^, *Km*^*r*^, *Inc*^*P*^, *tra*^*+*^	Shandong University, China
pJRD215	Sm^r^, Km^r^, IncQ, *mob*^+^	[12]
pSDU1	Cm^r^, IncQ, *mob*^+^	this study
pSDU1Δmob	Cm^r^, IncQ	this study
pSDU1ΔmobΔorfEF	Cm^r^, IncQ	this study

### PCR and DNA recombination technique

Premix PrimeSTAR HS DNA polymerase, restriction enzymes and T4 DNA ligase were purchased from Takara Co. and used in accordance with the manufacturer's instructions. Oligonucleotides sequences of primers used in this work were listed in Table 6.

**Table 6:** Primers used in this work.

Name	Sequence (5’ to 3’)^a^
Cm S1sen	GCGGTAGTTTATCACAGTTAAATTGCTAACGCAGTCAGGCACCGTGTATGGAGAAAAAAATCACTGG
CmS2 sen	CTGA*GAATTCATGCAT*TCATGTTTGACAGCTTATCATCCATAAGGTTTAATGCGGTAGTTTATCACAG
Cm ant	CTTC*GTGCAC*ATTCATCCGCTTATTATCACTT
P215 sen	CTTC*GTGCAC*TCCTTGCAATACTGTGTT
P215 ant	CTTC*ATGCAT*CCC*AAGCTT*AGAGCATACATCTGGAAGCA
MCS sen	CTAG*TCTAGA*GGATCCCCGGGTACCGAGCTCGAATTCATGCATTCATGTTTGACAGCT
MCS ant	CTAG*TCTAGA*GTCGACCTGCAGGCATGCAAGCTTAGAGCATACATCTGGAAGCA
RepB S1 sen	TGGAGGCACAGCATTGAGCCGAAAAGCAAAAGCAACAGCGAGGCAGCATGAAGAACGACAGGACT
RepB S2 sen	TCCCCTTAACCATCTTGACACCCCATTGTTAATGTGCTGTCTCGTAGGCTATCATGGAGGCACAGCATTG
Remove mob ant	GCTGAATGATCGACCGAGAC
orfEF sen	ATGGCTACCCATAAGCCTATCAAT
orfEF ant	AAAACCCCCTTCTGTGCGTGAGT
RepA sen	CGGGTGCTCTATCGTGTTCCTG
RepA ant	GCGGATGTTATCGACCAGTACC
tetH sen	ACGGCGTTAAGGAAGCACTG
tetH ant	GTCGTCACTTTCGGCATAGA
tetH Xba sen	CTAG*TCTAGA*ACGGCGTTAAGGAAGCACTG
tetH Hind ant	TCCC*AAGCTT*GTCGTCACTTTCGGCATAGA

^a^ Artificial restriction sites are italic and underlined.

### Plasmid construction

As shown in Fig 2, a P2-*cat* fragment (842 bp) was generated by two rounds of PCR. In the first round, PCR amplification was performed using pACBSR as the template and Cm S1sen and Cm ant primers. The generated PCR product (791 bp) was used as the template for the second round of PCR amplification using primers CmS2 sen and Cm ant. The P2 promoter sequence was introduced into the PCR fragments by primers CmS1 sen and CmS2 sen. The P2-*cat* fragment was digested with *Alw*44 I and *EcoT*22 I, ligated to the same enzyme treated OriV-*mob*-*repB*-*orfEF*-*repA*-*repC* fragment (5,539 bp) amplified from pJRD215 using primers P215 sen and P215ant, producing a new plasmid (6,361 bp). Then, the generated plasmid was amplified by PCR using primers MCS sen and MCS ant, and the generated fragment (6,438 bp) was digested with *Xba* I and self-ligated to produce the plasmid pSDU1 that had the MCS from pUC19. To delete a part of *mob* region on pSDU1, two round PCR were carried out to construct the non-mobilizable plasmid pSDU1Δmob. The first-round PCR amplification was carried out using plasmid pSDU1 as template and with the primers (RepB S1 sen and Remove mob ant). The generated PCR product (4,840 bp) was used as the template for the second round PCR amplification using 5’end phosphorylated primers RepB S2 sen and Remove mob ant. For the phosphorylation of primers, the PCR fragment (4,894 bp) could be self-ligated, generating the plasmid pSDU1Δmob. Finally, the 5’end phosphorylated primers orfEFsen and orfEF ant were used for PCR amplification from pSDU1Δmob and the amplified fragment (4,444 bp) were self-ligated to construct pSDU1ΔmobΔorfEF. All the sequences of the constructed plasmids were verified by DNA sequencing.

The nucleotide sequences of pSDU1, pSDU1Δmob and pSDU1ΔmobΔorfEF have been deposited in NCBI GenBank database under accession number MF163397, MF163398, and MF163399, respectively.

### Gene transfer manipulations

The detailed steps of electroporation in *A*. *caldus* MTH-04 and the methods for the determination of transformation efficiency and frequency of plasmids were described earlier [11]. Conjugation of plasmids from *E*. *coli* to *A*. *caldus*, the reverse-mobilization of plasmids from *A*. *caldus* MTH-04 to *E*. *coli*, and determination of their mobilization frequencies in *A*. *caldus* were carried out in accordance with the reference [9].

### Quantification of plasmid copy number in *A*. *caldus*

The copy numbers of the plasmids in *A*. *caldus* MTH-04 cultivated in liquid Starkey-S^0^ medium were determined by SYBR-Green-based real-time qPCR according to the methods described in the references [29,33]. First, two primer sets specific to the tetrathionate hydrolase gene (*tetH*) and the *repA* gene were designed and denoted as the *tetH*- and *repA*- set, respectively. The sequences of the two sets of primers (RepA sen and RepA ant, tetH sen and tetH ant) are listed in Table 6. Second, a standard plasmid pJRD215-tetH, carrying part of *tetH* gene amplified from *A*. *caldus* MTH-04 chromosomal DNA using primers tetH Xba sen and tetH Hind ant, was constructed to confirm the amplification specificities and efficiencies of primers *tetH*-set and the *repA*-set. Third, real-time qPCR amplification and analysis were performed using a LightCycler instrument with software version 3.5 (Roche Diagnostics). SYBR *Premix Ex* Taq was purchased from Takara Co. The total DNA of *A*. *caldus* MTH-04 transformants was extracted using the QIAamp DNA Mini kit (Qiagen Co.). The thermal cycling protocol was as follows: initial denaturation for 2 min at 95°C followed by 40 cycles of 10 s at 95°C, 10 s at 55°C and 15 s at 72°C. The fluorescence signal was measured at the end of each extension step (72°C). After the amplification, a melting curve analysis with a temperature gradient of 0.5°C/s from 65 to 90°C was performed to confirm that only the specific products were amplified. Finally, the plasmid copy number in *A*. *caldus* was determined using absolute quantification and relative quantification methods [29,33].

### Stability analysis of plasmids in *A*. *caldus*

Stability analysis of plasmids in *A*. *caldus* in the absence of antibiotic was determined as follows: A single colony of *A*. *caldus* transformant was inoculated into 20 ml of Starkey-S^0^ liquid medium in the absence of any antibiotic, incubated at 40°C for 6 days; then 10% (v/v) of the fully grown cultures was transferred to 20 ml of a fresh Starkey-S^0^ medium and incubated at 40°C for 6 days; the resulting culture was transferred additional four times and an aliquot was taken at the beginning of each cycle, diluted and plated onto solid Starkey-Na_2_S_2_O_3_ medium both with and without antibiotic. Plasmid stability was calculated as a ratio of the number of colonies on medium with or without antibiotic. Similarly, stability analysis of plasmids in *A*. *caldus* with the existence of antibiotic was tested according to the above manipulations except that the culturing media was replaced by the antibiotics containing liquid media.
